# Risk factors and protective measures for desmoid tumours in familial adenomatous polyposis: retrospective cohort study

**DOI:** 10.1093/bjsopen/zrae148

**Published:** 2024-12-30

**Authors:** Emanuele Rausa, Valeria Duroni, Davide Ferrari, Stefano Signoroni, Chiara M Ciniselli, Sara Lauricella, Clorinda Brignola, Maria T Ricci, Alessandro Gronchi, Paolo Verderio, Marco Vitellaro

**Affiliations:** Unit of Hereditary Digestive Tract Tumours, Fondazione IRCCS Istituto Nazionale dei Tumouri, Milan, Italy; Colorectal Surgery Division, Fondazione IRCCS Istituto Nazionale dei Tumouri, Milan, Italy; Unit of Bioinformatics and Biostatistics, Fondazione IRCCS Istituto Nazionale dei Tumouri, Milan, Italy; Unit of Hereditary Digestive Tract Tumours, Fondazione IRCCS Istituto Nazionale dei Tumouri, Milan, Italy; Colorectal Surgery Division, Fondazione IRCCS Istituto Nazionale dei Tumouri, Milan, Italy; General Surgery Residency Programme, Università degli Studi di Milano, Milan, Italy; Unit of Hereditary Digestive Tract Tumours, Fondazione IRCCS Istituto Nazionale dei Tumouri, Milan, Italy; Unit of Bioinformatics and Biostatistics, Fondazione IRCCS Istituto Nazionale dei Tumouri, Milan, Italy; Unit of Hereditary Digestive Tract Tumours, Fondazione IRCCS Istituto Nazionale dei Tumouri, Milan, Italy; Colorectal Surgery Division, Fondazione IRCCS Istituto Nazionale dei Tumouri, Milan, Italy; Unit of Hereditary Digestive Tract Tumours, Fondazione IRCCS Istituto Nazionale dei Tumouri, Milan, Italy; Unit of Hereditary Digestive Tract Tumours, Fondazione IRCCS Istituto Nazionale dei Tumouri, Milan, Italy; Sarcoma Surgery Unit, Department of Surgery, Fondazione IRCCS Istituto Nazionale Dei Tumouri, Milan, Italy; Unit of Bioinformatics and Biostatistics, Fondazione IRCCS Istituto Nazionale dei Tumouri, Milan, Italy; Unit of Hereditary Digestive Tract Tumours, Fondazione IRCCS Istituto Nazionale dei Tumouri, Milan, Italy; Colorectal Surgery Division, Fondazione IRCCS Istituto Nazionale dei Tumouri, Milan, Italy

## Abstract

**Background:**

Familial adenomatous polyposis is a cancer-predisposing syndrome caused by germline pathogenic variants of the adenomatous polyposis coli gene, leading to numerous colorectal polyps and a high risk of colorectal cancer. Desmoid tumours have become significant in the management of familial adenomatous polyposis after a colectomy, yet the exact incidence remains undetermined due to a lack of dedicated surveillance.

**Methods:**

This retrospective study accessed data from the prospectively maintained Hereditary Digestive Tumours Registry from 2000 to 2023. Desmoid-free survival was analysed using Cox regression and Kaplan–Meier curves.

**Results:**

A total of 202 patients with familial adenomatous polyposis who underwent colorectal surgery were enrolled. Of the patients, 21 (10.4%) developed intra-abdominal desmoid tumours after surgery. Desmoid tumours were associated with surgical procedure, histology of cancer at the time of surgery, and family history of intra-abdominal desmoid tumours. The overall desmoid-free survival probability at a median follow-up of 84 months was 90%. Histology of cancer at the time of surgery (HR 0.25 (95% c.i. 0.10 to 0.59)), family history of intra-abdominal desmoid tumours (HR 2.92 (95% c.i. 1.22 to 6.97)), an open approach compared with a laparoscopic approach (HR 2.43 (95% c.i. 1.03 to 5.73)), and a proctocolectomy compared with a rectal-sparing total colectomy (HR 3.01 (95% c.i. 1.28 to 7.10)) emerged as significant prognostic factors affecting desmoid-free survival.

**Conclusion:**

A minimally invasive rectal-sparing total colectomy appears protective against the development of desmoid tumours. Early surgery does not seem to increase desmoid tumour risk. A dedicated surveillance regimen for desmoid tumours in patients with familial adenomatous polyposis is needed to improve outcomes and quality of life.

## Teaser

This study investigated risk factors for the development of desmoid tumours in patients with familial adenomatous polyposis after prophylactic colorectal surgery. The findings suggest that minimally invasive rectal-sparing surgery and non-malignant histology can protect against desmoid tumours. A dedicated surveillance regimen is needed to improve the early detection and management of desmoid tumours in patients with familial adenomatous polyposis.

## Introduction

Familial adenomatous polyposis (FAP) is a cancer-predisposing syndrome, with autosomal dominant inheritance, caused by germline pathogenic variants of the adenomatous polyposis coli (*APC*) gene^1^. This complex genetic disorder is characterized by the formation of numerous (greater than 100) colorectal adenomatous polyps, carrying an almost certain risk of developing colorectal cancer (CRC) throughout a patient’s lifetime^2^. Since the introduction of preventive colectomy to reduce the incidence of CRC, typically recommended between the ages of 20 and 25 years, desmoid tumours (DTs) have gained growing importance in the management of FAP^3^. DTs are benign myofibroblastic proliferations that exhibit heterogeneous growth patterns, ranging from aggressive to stable or, eventually, to spontaneous resolution^4^. It has been estimated that DTs are 800–1000 times more common in FAP individuals than in the general population, but the exact incidence has not been determined due to the lack of a dedicated surveillance regimen^5^. Retrospective studies have shown that 10–25% of patients with FAP develop at least one DT during their lifetime, particularly in an intra-abdominal/retroperitoneal context^6^. Previous studies have shown that potential non-modifiable risk factors for FAP-associated DTs are a positive family history, specific genotypes, and previous abdominal surgery^7^. Additionally, Church *et al*.^8^ demonstrated that mutations occurring 3′ to codon 1399 are linked to a more symptomatic and severe disease presentation. Abdominal surgery is also widely accepted as a precipitating factor leading to the development or progression of DTs^9^. The aim of the present study was to assess risk factors for the development of DTs in patients with FAP after prophylactic colorectal surgery at a quaternary referral centre for hereditary CRC. These findings will be considered as a basis to design a dedicated surveillance protocol for FAP-related DTs.

## Methods

### Study sample

Institutional Review Board approval (INT 191/19) was obtained and data from the prospectively maintained Hereditary Digestive Tumours Registry at the Fondazione IRCCS Istituto Nazionale dei Tumouri of Milan (Italy) were retrospectively accessed from 2000 (coinciding with the establishment of the registry at the Fondazione IRCCS Istituto Nazionale dei Tumouri of Milan) to 2023 for this retrospective observational cohort study. The primary purpose of this registry is to prospectively collect and maintain comprehensive clinical and genetic data on patients with hereditary gastrointestinal cancer syndromes, including FAP, for ongoing research and clinical management purposes. Written informed consent for research purposes is routinely obtained from patients included in the Hereditary Digestive Tumour Registry at the time of their inclusion. All patients with FAP, with a germline pathogenic variant of *APC*, who underwent colorectal surgery (that is a proctocolectomy or a total colectomy with rectal sparing) at the Fondazione IRCCS Istituto Nazionale dei Tumouri of Milan to prevent or treat CRC were enrolled in the present study. *APC* germline variants were determined from blood samples using gene sequencing and/or multiplex ligation-dependent probe amplification techniques. *APC* germline variants were classified as pathogenic or likely pathogenic following the guidelines of the American College of Medical Genetics and Genomics^10^. Patients were regularly followed up at the outpatient clinic according to the National Comprehensive Cancer Network guidelines for FAP^3^. Patients who had diagnoses of intra-abdominal DTs before colorectal surgery were excluded.

### Statistical analysis

Characteristics of the study sample are summarized using descriptive statistics and frequency distribution tables; associations between the presence of intra-abdominal DTs and clinical, pathological, and genetic characteristics were assessed using a chi-squared test or Fisher’s exact test, as appropriate, whereas the non-parametric Wilcoxon test was used for continuous variables^11^. The pattern of desmoid-free survival (DFS) was assessed using univariate and bivariate Cox regression models^12,13^. DFS was defined as the time from the date of surgery to the date of DT occurrence or of the last follow-up. The results are reported in terms of HR (95% c.i.) and the pattern of DFS is depicted using Kaplan–Meier curves. The relationship between age at surgery and body mass index (BMI) (on a continuous scale) was investigated by resorting to a regression model based on restricted cubic splines^13^. For each bivariate model, the predictive capacity was assessed using the C-statistic and its 95% c.i., as described by Uno *et al*.^14^, as a measure of model performance. A model was considered to have statistically significant performance when the lower boundary of the 95% c.i. for the C-statistic exceeded 0.50. All statistical analyses were performed using SAS^®^ Studio (SAS Institute, Inc., Cary, NC, USA; version 5.2), adopting a nominal α level of 5%. Graphical representations were obtained using R software (R Foundation for Statistical Computing, Vienna, Austria), with the ‘ggplot2’ and ‘survminer’ packages.

## Results

A total of 202 patients with FAP, with a confirmed germline pathogenic variant of *APC*, were enrolled in the present study and, of these patients, 21 (10.40%) developed an intra-abdominal DT after abdominal surgery. The clinical, pathological, and genetic characteristics of the cohort are summarized in *Table 1*. Among the 21 DTs, 15 were intra-abdominal and 6 were located in the abdominal wall. The median length of the major axis was 5 (range 2–30) cm and 16 patients were diagnosed because they were symptomatic. Males accounted for 53.47% of the patients, the median age of the patients was 25.5 (range 7–77) years, and the median BMI of the patients was 22 (range 15–40) kg/m^2^. More than a quarter of the patients (27.72%) had a family history of intra-abdominal DTs. The surgical specimen at the time of colectomy indicated that 14.85% of patients had an adenocarcinoma and that 32 patients had a ‘high-risk codon’ variant (codons 543–713 or 1440–2843 affected). The majority of the cohort underwent laparoscopic surgery and the majority of the cohort underwent a rectal-sparing total colectomy (76.73% and 73.27% respectively). Among the 54 patients who underwent a proctocolectomy, an ileal pouch-anal anastomosis (IPAA) was performed in 51 patients. The procedure was two staged in 48 patients. A pouch failure occurred in five patients and one of them developed a DT. A postoperative complication occurred in 6% of patients. A DT after surgery was significantly associated with the surgical procedure performed (a total colectomy *versus* a proctocolectomy, *P* = 0.005) and the surgical approach (laparoscopic *versus* open approach, *P* = 0.011). Histology of cancer at the time of surgery (adenocarcinoma *versus* low-grade dysplasia/high-grade dysplasia, *P* = 0.005) and family history of intra-abdominal DTs (*P* = 0.008) were found to be associated with a DT after surgery.

**Table 1:** Clinical, pathological, and genetic characteristics of the study patients, according to the presence or absence of an intra-abdominal desmoid tumour, as well as overall

	Intra-abdominal desmoid tumour (*n* = 21)	No intra-abdominal desmoid tumour (*n* = 181)	Study cohort (*n* = 202)
**Sex**			
Male	11 (52.38)	97 (53.59)	108 (53.47)
Age (years), median (range)	27 (14–61)	25 (7–77)	25.5 (7–77)
BMI (kg/m^2^), median (range)	22 (16–29)	22 (15–40)	22 (15–40)
**Histology of cancer at the time of surgery**			
Adenocarcinoma	8 (38.10)	22 (12.15)	30 (14.85)
Low-grade dysplasia/high-grade dysplasia	13 (61.90)	159 (87.85)	172 (85.15)
**Family history of intra-abdominal desmoid tumours**			
Yes	11 (52.38)	45 (24.86)	56 (27.72)
No	10 (47.62)	135 (74.59)	145 (71.78)
Missing	0 (0)	1 (0.55)	1 (0.50)
**Surgical approach**			
Laparoscopic	11 (52.38)	144 (79.56)	155 (76.73)
Open	10 (47.62)	37 (20.44)	47 (23.27)
**Surgical procedure**			
Total colectomy	10 (47.62)	138 (76.24)	148 (73.27)
Proctocolectomy	11 (52.38)	43 (23.76)	54 (26.73)
**Number of abdominal surgeries**			
One	13 (61.90)	144 (79.56)	157 (77.72)
Two	7 (33.33)	34 (18.78)	41 (20.30)
Missing	1 (4.76)	3 (1.66)	4 (1.98)
**Postoperative complication**			
No	20 (95.24)	170 (93.92)	190 (94.06)
Yes	1 (4.76)	11 (6.08)	12 (5.94)
**Codons**			
High-risk codons*	3 (14.29)	29 (16.02)	32 (15.84)
Low-risk codons†	16 (76.19)	152 (83.98)	168 (83.17)
Large deletions‡	2 (9.52)	0 (0)	2 (0.99)

Values are *n* (%) unless otherwise indicated. *Includes codons 543–713 and 1440–2843. †Includes codons not included in the ‘high-risk codons’ group. ‡Comprises both ‘high-risk codons’ and ‘low-risk codons’.

### Prognostic value

**Fig. 1:**
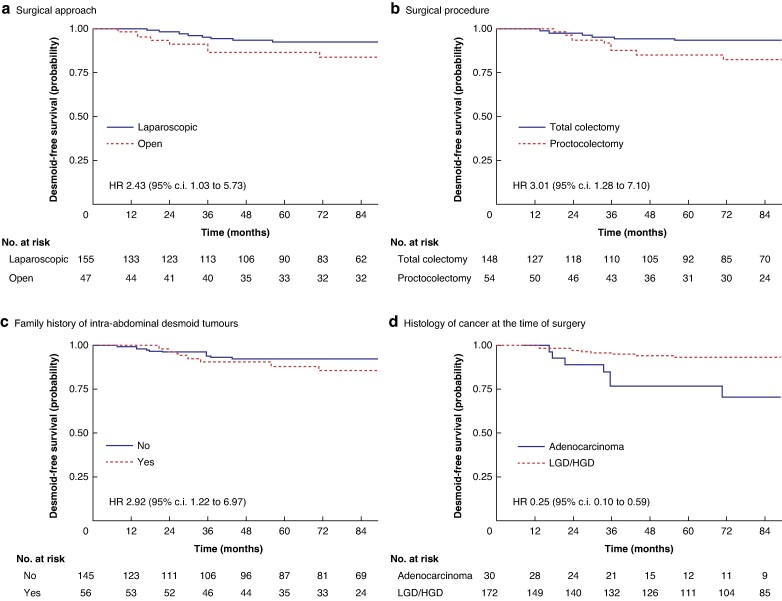
Probability of desmoid-free survival over 7 years **a** Surgical approach. **b** Surgical procedure. **c** Family history of intra-abdominal desmoid tumours. **d** Histology of cancer at the time of surgery. LGD/HGD, low-grade dysplasia/high-grade dysplasia.

The overall DFS probability at a median follow-up of 84 (interquartile range 48–136) months was 90% (95% c.i. 84% to 94%). *Table 2* summarizes the results of the univariate Cox regression model and *Fig. 1* depicts the significant variables in terms of Kaplan–Meier curves. Univariate Cox analysis revealed an association between DFS and surgical approach (HR 2.43 (95% c.i. 1.03 to 5.73)), surgical procedure (HR 3.01 (95% c.i. 1.28 to 7.10)), family history of intra-abdominal DTs (HR 2.92 (95% c.i. 1.22 to 6.97)), and histology of cancer at the time of surgery (HR 0.25 (95% c.i. 0.10 to 0.59)). An open surgical approach, a proctocolectomy procedure, a family history of intra-abdominal DTs, and an adenocarcinoma at the time of surgery demonstrated worst DFS (*Fig. 1*). Due to the associations observed between the significant variables in the univariate Cox analysis for DFS and considering the number of events per variable (EPV), only bivariate models were pursued to avoid multicollinearity. *Table 3* presents the results of the bivariate models, incorporating family history of intra-abdominal DTs alongside surgery-related information (such as type of surgical procedure) or histology of cancer at the time of surgery. Notably, all covariates included in the models maintained their statistical significance in the bivariate analysis. All models demonstrated a satisfactory capacity to predict DFS, with a C-statistic value greater than 0.70.

**Table 2:** Univariate Cox regression model assessing factors associated with desmoid-free survival; study cohort *n* = 202

	HR (95% c.i.)
**Sex**	
Male *versus* female	0.76 (0.32,1.80)
**Age (years)**	
Continuous	1.02 (0.98,1.05)
**BMI (kg/m^2^)**	
Continuous	0.95 (0.85,1.06)
**Histology of cancer at the time of surgery**	
Low-grade dysplasia/high-grade dysplasia *versus* adenocarcinoma	0.25 (0.10,0.59)
**Family history of intra-abdominal desmoid tumours**	
Yes *versus* no	2.92 (1.22,6.97)
**Surgical approach**	
Open *versus* laparoscopic	2.43 (1.03,5.73)
**Surgical procedure**	
Proctocolectomy *versus* total colectomy	3.01 (1.28,7.10)

**Table 3:** Bivariate Cox regression models assessing factors associated with desmoid-free survival

	HR (95% c.i.)	C-statistic (95% c.i.)
**Model 1**		
Family history of intra-abdominal desmoid tumours	3.11 (1.29,7.46)	0.78 (0.67,0.90)
Surgical approach	2.58 (1.09,6.11)	
**Model 2**		
Family history of intra-abdominal desmoid tumours	2.78 (1.16,6.67)	0.78 (0.68,0.88)
Surgical procedure	2.93 (1.24,6.93)	
**Model 3**		
Family history of intra-abdominal desmoid tumours	3.39 (1.40,8.23)	0.78 (0.69,0.88)
Histology of cancer at the time of surgery	0.21 (0.09,0.52)	

## Discussion

This study’s findings suggest that both non-malignant histology and rectal-sparing minimally invasive surgery can serve as protective factors influencing DFS. Additionally, the histology of cancer at the time of surgery and a family history of intra-abdominal DTs appear to be critical risk factors, regardless of the specific germline variant. A rectal-sparing total colectomy and a laparoscopic approach seem to be protective factors against the development of DTs. No significant differences between the sexes were found. The prevalence of DTs in the cohort was 10.4%, consistent with most previously published series. However, the largest series in the literature, by Sommovilla *et al*.^15^, reported an incidence of DTs of 28.9%, possibly related to a higher rate of open surgical procedures, which was greater than 50%.

Despite surgery being a well-known risk factor for developing *de novo* DTs, a prophylactic colectomy remains the preferred treatment to prevent CRC in patients with FAP^6^. However, the optimal timing and best surgical procedure (a total colectomy with an ileorectal anastomosis *versus* a proctocolectomy with an ileoanal anastomosis) are still debated^7^. The present findings did not show a significant association between DFS and age at the time of prophylactic surgery (*Fig. S1*). It can be speculated that performing preventive colorectal surgery earlier would not increase the risk of developing a DT compared with delaying the surgery.

Currently, conflicting evidence remains regarding the differences in the prevalence of *de novo* DTs after either a total colectomy with an ileorectal anastomosis or a restorative proctocolectomy with an ileoanal anastomosis^16^. Gega *et al*.^17^ observed that *de novo* DTs arose in 12% and 13% of patients after a restorative proctocolectomy and an ileorectal anastomosis respectively. In contrast, Sommovilla *et al*.^15^ suggested that a proctocolectomy and an ileal pouch had a higher risk of DTs than an ileorectal anastomosis, regardless of the surgical approach. Interestingly, a pioneering study comparing laparoscopic surgery with open surgery showed that the minimally invasive approach dramatically decreases the risk of DTs after a prophylactic colectomy^18^.

In contrast to sporadic DTs, for which optimal management has been determined over the past 10 years, the treatment of DTs in patients with FAP still has several unresolved areas that need optimization^5,19^. This is particularly important, as *APC*-related DTs are described as more clinically aggressive and harder to treat. The spectrum and onset of symptoms depend on the features, site, size, and number of DT lesions^20^.

Specifically, FAP-related DTs are mostly found in an intra-abdominal/retroperitoneal context and may cause intestinal obstruction or perforation, ureter compression, ischaemic lesions, abscess formation, and digestive haemorrhage. Identified risk factors for FAP-associated DTs include surgical trauma, a positive family history, mutation sites beyond codon 1309 (especially at the 5′ end of codon 1444), the influence of oestrogen, pregnancy, and female sex^21,22^.

Historically, complete surgical excision with clear margins was considered optimal, particularly for extra-abdominal and abdominal wall DTs. Some have questioned the impact of clear margins and adjuvant therapy on the risk of recurrence or the development of *de novo* DTs^22^. Interestingly, Penel *et al*.^23^ compared the outcomes of non-operative treatment with those of immediate radical surgery and found that the 2-year relapse-free survival rate was similar for the two groups. Currently, active surveillance is usually preferred for FAP-associated DTs, especially for intra-abdominal DTs. Surgical excision attempts for DTs involving the small bowel, mesentery, and great vessels often lead to major resections and complications, such as ischaemia, fistulas, and bowel obstruction^24^. Intra-abdominal DTs tend to recur even after complete surgical resection, with a mean time to recurrence of approximately 18 months (ranging from 4 months to 12 years), and a second procedure is required for 75–85% of patients^25^. The European Society for Medical Oncology guidelines and the global DT consensus recommend starting with active surveillance or medical therapy for advanced DTs, depending on the symptoms^26^. It would be beneficial if the surveillance regimen could detect intra-abdominal DTs before excessive growth or the onset of abdominal symptoms. Given the rarity of the disease and the difficulty in identifying small intra-abdominal lesions, it is recommended to conduct active surveillance with MRI every 6–12 months, starting from the time of prophylactic surgery. However, the existing literature is insufficient to fully support these recommendations and further multicentre prospective studies are needed^27^. Finally, diagnosing a DT typically relies on a percutaneous biopsy. However, in challenging anatomical locations (for example intra-abdominal locations), a biopsy may require a surgical approach. In patients with FAP who have undergone preventive surgery, minimizing further surgical trauma is advisable, as it could potentially promote DT growth and harm the remaining small bowel^9,28^. In specialized centres, medical treatment can be considered even without histological confirmation^5^.

This study has limitations, including its retrospective design. There is an absence of a dedicated surveillance regimen for DTs in patients with FAP and therefore intra-abdominal DTs are only identified when patients become symptomatic. This factor could bias the timing of DT detection or potentially lead to an underestimation of the true incidence of these tumours in this patient cohort. The relatively small sample size and the pairwise associations between the investigated factors limited the ability to perform a comprehensive multivariate analysis of the factors affecting DFS. Finally, the study did not collect detailed information on specific surgical techniques, such as the type of anastomosis or the use of a temporary ileostomy, which could potentially influence the risk of DT development. Future prospective studies with more granular data collection on surgical techniques may provide further insights into the relationship between surgical factors and the occurrence of DTs in patients with FAP.

In conclusion, minimally invasive approach and total colectomy with rectal sparing appear to independently offer protection against DT development. While no optimal surgical approach has been definitively established, early surgery does not seem to increase DT risk. A dedicated surveillance regimen for DTs in FAP patients is needed to facilitate early detection and enable timely intervention, potentially improving patient quality of life by identifying and managing DTs before they become symptomatic or cause complications.

## Supplementary Material

zrae148_Supplementary_Data

## Data Availability

The data presented in this study are available on request from the corresponding author.
