# Supplementary material for: Risk factors and protective measures for desmoid tumours in familial adenomatous polyposis: retrospective cohort study
Source: BJS Open. 2024 Dec 30;9(1):zrae148. doi: 10.1093/bjsopen/zrae148 (PMC11683362; doi:10.1093/bjsopen/zrae148)
Supplement: zrae148_Supplementary_Data [file zrae148_supplementary_data.docx]

**Risk Factors and Protective Measures for Desmoid Tumours in Familial Adenomatous Polyposis: Retrospective cohort study**

**Emanuele Rausa** [^#^](https://pubmed.ncbi.nlm.nih.gov/38849509/#full-view-equal-contrib-explanation)**^a,b^, Valeria Duroni**  [^#^](https://pubmed.ncbi.nlm.nih.gov/38849509/#full-view-equal-contrib-explanation)**^c^, Davide Ferrari^a,b,d^, Stefano Signoroni^a^, Chiara Maura Ciniselli^c^, Sara Lauricella^a,b^, Clorinda Brignola^a^, Maria Teresa Ricci^a^, Alessandro Gronchi^e^, Paolo Verderio^c^, Marco Vitellaro^a,b^**

^a^ Unit of Hereditary Digestive Tract Tumours, Fondazione IRCCS Istituto Nazionale dei Tumouri, Milan, Italy

^b^ Colorectal Surgery Division, Fondazione IRCCS Istituto Nazionale dei Tumouri, Milan, Italy

^c^ Unit of Bioinformatics and Biostatistics, Fondazione IRCCS Istituto Nazionale dei Tumouri, Milan, Italy

^d^ General Surgery Residency Program, Università degli Studi di Milano, Milan, Italy.

^e^ Sarcoma Surgery Unit, Department of Surgery, Fondazione IRCCS Istituto Nazionale Dei Tumouri, Milan, Italy.

[^#^](https://pubmed.ncbi.nlm.nih.gov/38849509/#full-view-equal-contrib-explanation) Contributed equally as co-first author.

**Corresponding author.** Davide Ferrari, davide.ferraro@istitutotumori.mi.it **ORCID ID 0000-0002-7058-5011**; **Twitter** @DFerrariMD; @Signoroni; @lauricella_sara; @cloribri; @alegronchi

**Supplementary Materials - Index**

| **Supplementary Figures and Tables** |  |
| --- | --- |
| Supplementary Figure 1 | *page 2* |
|  |  |

**Supplementary Figures and Tables**

**Supplementary Figure 1**. Seven-year Desmoid Free Survival (DFS) probability curves of age at surgery stratify for surgical procedure. The curve depicts the predicted DFS probability, at the median follow-up time, of age considered on its continuous scale in patients who underwent (a) total colectomy and (b) proctocolectomy with detail on histology of cancer represented by the coloured dots.


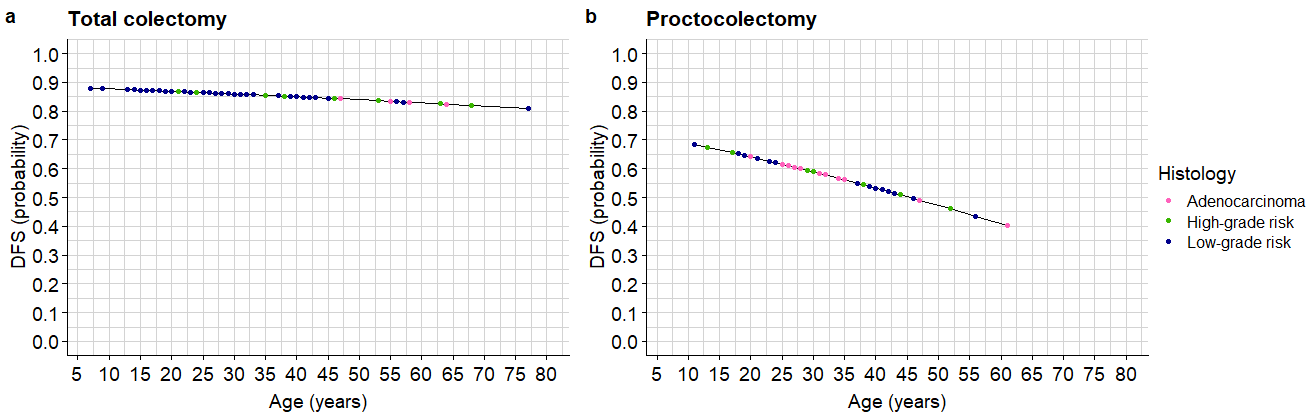


**References**
